# Baby pangolins on my plate: possible lessons to learn from the COVID-19 pandemic

**DOI:** 10.1186/s13002-020-00366-4

**Published:** 2020-04-21

**Authors:** Gabriele Volpato, Michele F. Fontefrancesco, Paolo Gruppuso, Dauro M. Zocchi, Andrea Pieroni

**Affiliations:** University of Gastronomic Sciences of Pollenzo, Piazza V. Emanuele II, I-12042 Bra/Pollenzo, Italy

## Abstract

The Journal of Ethnobiology and Ethnomedicine (JEET), throughout its 15 years of existence, has tried to provide a respected outlet for scientific knowledge concerning the inextricable links between human societies and nature, food, and health. Ethnobiology and ethnomedicine-centred research has moved at the (partially artificial and fictitious) interface between *nature and culture* and has investigated human consumption of wild foods and wild animals, as well as the use of wild animals or their parts for medicinal and other purposes, along with the associated knowledge, skills, practices, and beliefs. Little attention has been paid, however, to the complex interplay of social and cultural reasons behind the increasing pressure on wildlife. The available literature suggest that there are two main drivers that enhance the necessary conditions for infectious diseases to cross the species barrier from wild animals to humans: (1) the encroachment of human activities (e.g., logging, mining, agricultural expansion) into wild areas and forests and consequent ecological disruptions; and, connected to the former, (2) the commodification of wild animals (and natural resources in general) and an expanding demand and market for wild meat and live wild animals, particularly in tropical and sub-tropical areas. In particular, a crucial role may have been played by the *bushmeat-euphoria* and attached elitist gastronomies and conspicuous consumption phenomena. The COVID-19 pandemic will likely require ethnobiologists to reschedule research agendas and to envision new epistemological trajectories aimed at more effectively mitigating the mismanagement of natural resources that ultimately threats our and other beings’ existence.

*In memory of Dr. Javier Caballero, Autonomous University of Mexico and JEET board member, who passed away 12 March 2020.*

## Ethnobiology, gastronomy, and COVID-19

The COVID-19 pandemic poses to the scientific community and to its worldwide audience important open research questions in ethnobiology and ethnomedicine. Questions that have regularly reappeared during the past century with the spread of the various pathogenic viruses originally derived from animals (e.g., Spanish flu, Asian flu, AIDS, Nipah, Marburg, swine flu, SARS, MERS, and Ebola):

- Why does the intensification of the use of certain animal resources happen in certain places at certain times?
- Is this intensification happening in specific areas during a particular period due to commodification of Traditional/Indigenous/Local Ecological Knowledge (TILEK) or to which other socio-cultural factors?
- Is the search for an exclusive, elitist gastronomy “to blame”?
- How can we prevent such unsustainable intensifications?

The Journal of Ethnobiology and Ethnomedicine (JEET), throughout its fifteen years of existence, has tried to provide a respected outlet for scientific knowledge concerning the inextricable links between human societies and nature, food, and health. It has specifically covered these relationships from an ethno-scientific perspective, thus focusing on the complex systems of TILEK and their transformations across time and space.

In the past few decades, ethnobiology and ethnomedicine-centred research has moved at the (partially artificial and fictitious) interface between *nature and culture* and has tried to investigate the socio-cultural contexts in which domesticated and “wild” species and their ecosystems are perceived, used, and managed.

Specifically, JEET has published numerous papers addressing human consumption of wild foods and wild animals, as well as papers addressing the use of wild animals or their parts for medicinal and other purposes, along with the associated knowledge, skills, practices, and beliefs; less attention has been paid, however, to the reasons behind the intensification of the use of certain natural resources and, especially, to the links between their commodification and the emergence of new diseases from wildlife.

Zoonotic diseases constitute about 70% of all known emerging diseases and are Swords of Damocles hanging over global public health [1–3]. SARS-CoV-2 is the latest of several viruses that have emerged in wildlife, crossed the species barrier from animals (e.g., bats, civets, pangolins, apes) to humans, mutated, and then spread from human to human. These diseases often have multiple animal reservoirs and intermediate hosts as well as complex transmission pathways, but viral transmission often requires direct or indirect contact between humans and animals.

A number of environmental and socio-economic factors are increasing contact rates between humans and wildlife: trade in wild animals for food and medicine, encroachment of humans and domestic animals into wildlife habitats, intensification of food systems and changes in land use in tropical and subtropical areas, globalization of agriculture and commerce, commodification of biodiversity and its traditional use, and consumption of bushmeat [4–6].

The complexities of the ecological, social, and economical dynamics of disease emergence, therefore, require interdisciplinary approaches, for which ethnobiology and human ecology are extremely well positioned. Given the key role that the dynamics occurring at the interface between the “wild” and the “domestic” have in the emergence of zoonotic diseases, ethnosciences need to reflect on the ongoing COVID-19 pandemic, its drivers, and implications. Many dozens of scholars have long investigated this interface both in terms of the dynamic relationships (based on knowledge, practices, rituals) that humans establish with the other living creatures and with local ecologies, and in terms of the impacts that human activities have on these ecologies and on other “webs of life.”

Because foods, food systems and food cultures play a key role in the emergence of zoonotic diseases, food studies explored through a truly trans-disciplinary gastronomic sciences-centred lens can further help to understand the processes and dynamics behind the consumption of “wild” animals, its commodification, and the system of beliefs and values that underpins it.

Here, we briefly discuss the COVID-19 pandemic within the broader socio-cultural and gastronomic context in which it originated and occurs now. First, we lay out the main human ecological drivers for increased contact between humans and (other) animals and the potential viral spillover: anthropogenic disturbance of forest ecosystems and increasing demand for meat and medicine derived from wildlife. We further discuss the reasons for the increased demand for bushmeat, both as a response to food insecurity and as a response to a demand for exclusive, elitist consumption. We then address the relations that occur between the commodification of wild animals and the traditional systems of knowledge and practices that have sustained continuity in wild animal use. Finally, we reflect on the ways in which COVID-19 relates to the Anthropocene idea, with the processes of intensification and commodification as underlying common drivers.

## Bats: an exemplary case study

In order to prefigure these dynamics (and the line of argument below), we use bats as an example because their situation illustrates clearly the complex relationships between emerging zoonotic diseases, the intensification and commodification of wild animals, and the key role of foods and food systems in this emergence. Bats have been hunted for food and medicine since pre-historic times in all inhabited continents, especially in the Asia-Pacific region where big fruit bats of the genus *Pteropus* represent an important food source for some populations as well as an important element of local gastronomy, and are considered a delicacy in many places [7].

Bat meat is cooked in various ways, such as fried, roasted, stewed, grilled, and stir-fried [8, 9]. Moreover, minced meat and whole bats cooked in hot pot (simmering flavored broth in which raw ingredients are cooked) are available in restaurants in Southern China [10]. The culinary use of bat meat is also widespread in other Southeast Asian and Pacific countries. In the Republic of Palau, whole fruit bats are boiled in a soup made with ginger, coconut milk, vegetables, and various spices. The dish is served in local restaurants [11]. In the Marianna Islands, the Chamorro people consider fruit bat, locally known as fanihi, a delicacy which they serve during social happenings. Bats are washed and cooked in a soup, and all parts, including the fur, viscera, and wing membranes are eaten [12].

Bats are also reservoirs of several viruses that can cause human diseases, including Nipah, Hendra, SARS, and probably MERS, Ebola, and COVID-19 as well [13–18].

Cross-species transmission from bats to humans can be direct (through contact with infected bats or their excreta) or indirect through intermediate hosts (e.g., civets for SARS, camels for MERS, perhaps pangolins for COVID-19 [19]). The SARS coronavirus, for example, was traced by Chinese scientists to cave-dwelling horseshoe bats in Yunnan Province, but in the market of Guangdong, China, where the epidemic originated, the virus was isolated from masked civets (*Paguma* sp.), which acted as intermediate hosts [20, 21].

In the last few decades, with increasing intensification of land use (e.g., logging, plantations agriculture) in areas where fruit bats live and with the commodification and widespread trade of live bats and bat meat, the ecology of fruit bats has been disrupted, as has the ecology of their viruses. Processes of land use change toward intensification have in many cases led to increased contact between fruit bats and domestic animals (e.g., while roosting in trees in and around livestock paddocks, feeding on fruits in orchards) and humans (e.g., bats drinking and urinating in open palm sap containers) as well as to increased opportunities for viral spillover. The disruption of bat ecology also results in increasing numbers of fruit bats seeking food in suburban and urban areas and increasing human and livestock contact with them or their fluids [22]. All this has largely increased the probability of viral spillover from bats to humans and/or to intermediate hosts (wild or domesticated) with which bats come into contact, with global connectivity then amplifying its human to human transmission. At the same time, the consumption of bats has spread to a wider pool of urban consumers, and in southern China bats are found regularly in markets [23], where they may be in cages in proximity to other wild animals. While bats were traditionally hunted and consumed within locally based and ecologically attuned systems of knowledge, and these systems of knowledge often have norms in place to avoid over-harvesting, the commodification of bats, as with many other wild animals, leads to a race for maximum extraction that will result in further loss of biodiversity, further loss of cultural diversity of all those populations relying on bats, and further disruption of the bat-dependent ecological cycles, with further ecological turbulence.

## Intensification of the use of wild animals: why does it happen?

Understanding the drivers and dynamics that underpin intensification and commodification processes are of tremendous importance. The available literature points to two main drivers that enhance the necessary conditions for viruses to cross the species barrier from wild animals to humans: (1) the encroachment of human activities (e.g., logging, mining, agricultural expansion) into wild areas and forests and consequent ecological disruptions; and, connected to the former, (2) the commodification of wild animals (and natural resources in general) and an expanding demand and market for wild meat and live wild animals, particularly in tropical and sub-tropical areas. The globalization of the world economy (high human population densities, global transport and movement of people, spreading of information via the internet, including gastronomic information and recipes involving wild animals) has sustained these drivers and facilitates human-to-human transmission.

The emergence of new zoonotic diseases in the last century has occurred mostly at the African and Asiatic frontiers between forest and urbanization/civilization. This can be understood as a reflection of the encroachment of human activities into forests and of the consequent disruption of local ecologies, including the ecology of viruses and their hosts. Indeed, changes in the ecology of reservoir species can have a great impact on the emergence of zoonotic diseases. Deforestation and urbanization have likely contributed to the emergence of the Ebola virus in West Africa. The encroachment of human activities into forests provides numerous paths for the transmission of viruses from bats to intermediate (including livestock) hosts. The Hindra viruses of East Australia originated from bats and horses sharing the same environment, i.e., a horse pasture. Bats adapted to roosting in trees in pastures after the forest in which they lived was logged and transformed to the point that it could no longer sustain bat populations. Similarly, the Nipah virus appeared in Malaysia in connection with a spike in intensive commercial pig husbandry, a condition that facilitated the transmission of the virus from the bat reservoir to a swine intermediate host, and from there to humans [24]. Bat populations, displaced by shrinking forests and forest ecosystems increasingly deprived of species, may turn to fruit orchards for food and roosting, thus increasing the chance of transmission to other animals and to humans when partially eaten fruits are subsequently consumed.

To the extent that humans transform and occupy the forest ecosystem (e.g., palm oil or tea plantations, livestock pastures), they disrupt the ecology of wild animals, which in turn may increase the likelihood of viruses finding their way into intermediate hosts (wild or domesticated) and eventually into humans. The MERS coronavirus, for example, appeared in Saudi Arabia in 2012, and has been shown to have bats as the original reservoir and camels as an intermediate host [25, 26]. Humans become infected after exposure to infected camels or consuming the raw milk and meat of camels. Although the dynamics of transmission from bats to camels are not yet understood, they may involve the increased contact that occurs between the two species in conditions of sedentary (versus nomadic) and stabled (versus open-air) camel husbandry, conditions in which bats could roost inside stables and spread viruses to the camels below with their urine, feces, and droplets.

## The bushmeat-euphoria

As subsistence needs and a globalized consumerist system pushes people (e.g., farmers, gatherers, and hunters, desperate for food and cash) into the forests, more is demanded and extracted from these areas, including wild animals used as food and medicine.

A diversity of local and seasonal wild animal-derived foods sustains the livelihood and economy of many American, African, and Asian communities. These products are materially and culturally important foods (e.g., providing nutrients, sustaining social cohesion, and cultural identity) as well as an integral part of the gastronomic basket of these communities. Wild food consumption, in many subsistence communities, is embedded into complex systems of traditional ethnobiological and ethnoecological knowledge about the species consumed, their biology and ecology, and ways of hunting, gathering or fishing, as well as traditional knowledge about processing, cooking, recipes and ways of consuming. Wild food consumption is also often entrenched into systems of beliefs, rituals, and taboos that aim to regulate communities’ engagement with wild natural resources and species.

In many parts of Africa, bushmeat (i.e., wild animals hunted/collected for food, such as mammals ranging from rodents to large species, reptiles) contributes substantially to the animal protein supply and often fetches a higher price in markets than livestock meat [27]. Roasted, boiled, smoked, or dried, bushmeat provides proteins and fat to rural and forest inhabitants, as well as cash from its commercialization [28]. The history of AIDS tells us today that HIV-1 and HIV-2 originated from SIV, a virus that was transmitted from non-human primates to humans in Central Africa at the beginning of the 20th century. The evidence that humans who participated in bushmeat hunting, trading, and butchering could easily acquire SIV, and that several transmissions of the virus from individual to individual in quick succession allowed it to mutate into HIV, is robust [29–31]. Some studies have postulated that high-risk transmission channels, allowing the virus to adapt to humans, emerged with colonialism and the growth of large African cities, in connection to a spread of prostitution [32, 33].

Bushmeat hunting is again on the rise today, particularly in those tropical and subtropical areas characterized by poverty and food insecurity. Hunters enter deep into forested areas following roads from logging and mining activities to source wild animals in response to a growing urban demand, with customers often regarding bushmeat as a delicacy and a prestige food. Indeed, it is not simply taste that is driving demand for bushmeat, as price, needs, familiarity, tradition, and prestige also play a role [34].

A striking example of the relationship between food insecurity and bushmeat hunting is provided by the lemurs of Madagascar. Borgerson et al. [35] have shown that most children in the households of wildlife hunters were malnourished. Bushmeat was often the only accessible food for these families, and under these circumstances, it is no wonder that hunters are lured into commercial bushmeat chains that provision hotel and restaurants with lemur meat as a prestige food [36]. Another study in Madagascar has predicted that the rate of childhood anaemia would increase 29% if access to bushmeat, including bat and lemur meat, was restricted, predominantly affecting the poorest households that could not afford to purchase meat from domesticated animals [37].

Poverty and food insecurity increase the demand for wild animals for consumption and trade, and thus contact between these animals and humans. Indeed, this is the socio-economic background for the Ebola and HIV epidemics. Interestingly, in a world that is ecologically and economically interconnected, causes and effects are complex and sometimes unexpected. It is therefore worth noting that in several parts of Africa the demand and consumption of bushmeat has increased as a consequence of the collapse of artisanal and small-scale fisheries due to industrial overfishing (from China, Korea, the EU) and fish population collapse along African coasts [38, 39]^1^.

At the same time, livelihoods are increasingly being commodified (i.e., dependent on products and services obtained with cash), and this commodification and the increasing need for cash drives further commodification of wild foods and animals formerly hunted and consumed for subsistence. This, connected with a demand for these foods in growing towns and cities, has driven additional extraction and the national and international trade of live animals and their meat [40, 41]. This all results in high demand for animal-derived products sold in formal and informal, rural and urban open-air markets as well as along streets and roadsides across many tropical and subtropical areas [42, 43]. The resulting market pressure on the species and on local communities often brings about the erosion of norms and taboos (e.g., regarding wild animal hunting and harvesting), the shifting of the economic value chain and of control over the resource from local producers to outsiders, the adoption of invasive technologies for harvesting, and an increase in wealth inequality within communities, thus threatening both social and environmental sustainability and resilience at multiple levels [41]. With increasing commodification of traditional and ecologically attuned systems of knowledge, these systems have often been bent to market imperatives for short-term gain, cheap resources, and cheap labor. Unsanctioned and poorly sanctioned processes of commodification (for some species all the way to wildlife farming) are threatening species previously consumed for subsistence, their population and habitat. For example, the mopane caterpillar, harvested from the mopane tree across southern Africa, has become a commodity sold in towns and cities as well as exported to Europe, and this has created stress and threats to local lives and livelihoods (as people witness the commodification of an important subsistence and seasonal resource), to the species itself (customary norms for sustainability discarded), to the mopane tree that hosts the caterpillar (trees are felled to reach caterpillars high up the canopy), and to the same savannah ecosystems of which the mopane tree is a keystone species (providing critical food to elephants, who in turn shape the ecosystem with their presence) [44]. With regard to mammals, the trade of live and recently slaughtered wild animals in “wet markets” (markets where live animals and freshly slaughtered meat are sold, and so named because of the large quantities of water used to slosh the floors) across many tropical and subtropical areas of the world (e.g., Peru, South-East Asia and China, Western Africa) has largely increased contact between different species of wild animals, and between them and humans. Much of this trade relates to the demand for products used in Traditional Chinese Medicine. Traditional Chinese Medicine makes large use of animal products, creating an environmental impact as well as health hazards [45]. Because this medicine is widespread and growing, there is increasingly higher demand for wildlife species and for the products obtained from them [46].

## Wild meat in elitist gastronomies

Over one century ago, Veblen [47] theorized that *conspicuous consumption*, i.e., the elitist consumption of expensive and superfluous foods and drinks, is one of the ways in which affluent classes flaunt their wealth and power. As Bourdieu [48] suggested, however, this strategy turns these products into a status symbol which is copied by other strata of society in search of legitimation. While this process intensifies the actual consumption of products, the “new rich” are the ones that are the most eager in mimicking [49]. This phenomenon is more than ever evident today, in a global society that is highly unequal and confers prestige to wealthier people [50], in particular in China; a country that more than others has experienced fast economic growth and the rise of new affluent social groups [51, 52]. While the new social status is generally marked by purchasing houses and luxury goods [53–55], food and foodways are also transformed. It is not just a matter of eating out in fine-dining restaurants [56], but rather asking for exclusive foods traditionally associated with the old elites [52], such as wild meat.

Asia is an epicenter for wildlife trafficking and wild animal consumption. In countries like China, Myanmar, Vietnam, and Thailand, the social status, prestige, and gastronomic exclusivity deriving from *ye wei* (literally “wild taste”) is the main driver of the demand for wild meat, particularly among the wealthiest and those aspiring to be. In the cuisine of Asian countries, *ye wei* refers to bushmeat and game including wild and exotic animals. Historically, members of the imperial courts in the dynastic eras used to request *ye wei*, including symbolic and magical animals or animal parts, for their meals. Nowadays, *ye wei* is widely sold in Asian wet markets, offered at restaurants, and requested by wealthy consumers because of their rarity and cost. In a recent survey conducted in China, almost a third of the respondents reported consuming wildlife, with consumers with higher incomes and higher education levels having higher wildlife consumption rates [57]. The rapid urbanization and shift to a market economy in these countries, and the subsequent emergence of hundreds of millions of potential middle-class consumers wanting to emulate elitist foodways, has boosted the demand for wild meat, trade of wild animals, attendance of wet markets, and food and medicinal consumption of wild animals. These animals are sourced legally or illegally, from the wild or from wildlife farms. A source for these species is the thousands of wildlife farms that have arisen in China during the last twenty years, which can be seen as attempts to intensify “wildlife production.” These farms raise a number of animals for food, from peacocks to porcupines and civets, which are often believed to have powerful medicinal and magical/symbolic properties. Indeed, the SARS coronavirus has been shown to use farmed civets as intermediate hosts before jumping to humans [58, 59]. Rhino horns, tiger bones, civet and pangolin meat, porcupines, bamboo rats, totoaba bladder, shark fins’ soup, and roasted bats are notable examples of this demand for wild luxury foods and/or medicinal items. Commercial chains run deep into forests to provision wealthy consumers by selling to restaurants, at “wet markets,” or through online platforms, where consumers can also find recipes and cooking advice. In recent years, the trade of wildlife for food and medicine has spread via the Internet, where virtual platforms and ecommerce websites sell wild animals or products obtained from them.

This demand is driving widespread legal and illegal trade of wild animals. Wildlife trafficking profits are estimated at $26 billion per year and are pushing many species (often critical for ecosystem functioning and resilience, and for the services these ecosystems provide to humans) towards extinction. Humans are literally eating and drinking species into extinction [60]. In these circumstances, wild meat commodification and its associated activities are likely to enhance the conditions for zoonotic infectious diseases to jump to humans, while global connectivity and human population density and movement then help to spread the virus from human to human.

## Pangolins: from medicinal item to exclusive delicacy

A prime example is the pangolin, the most trafficked animal in the world, which is the likely intermediate host of SARS-CoV-2 [61]. Pangolins are nocturnal insect-eating mammals living in the forests of Asia and Africa. Pangolins have long been hunted for food and traditional medicine across Asia and West and Central Africa [62, 63]. In Ghana, for example, people traditionally use different parts (scales, bones, head, and meat) for different purposes including spiritual protection, rheumatisms, infertility, and convulsions, while the meat was used for preparing charms for chiefs or tribal leaders [64]. In Sierra Leone, the scales, head, meat, and tail are prevalently used for food as well as for spiritual protection and to treat skin diseases and digestive problems [65]. Pangolins and their scales are similarly used (e.g., to ward off evil spirits and witchcraft) in Nigeria [66–68] and in Benin [69], as well as across India and Pakistan [70, 71].

**Fig. 1:**
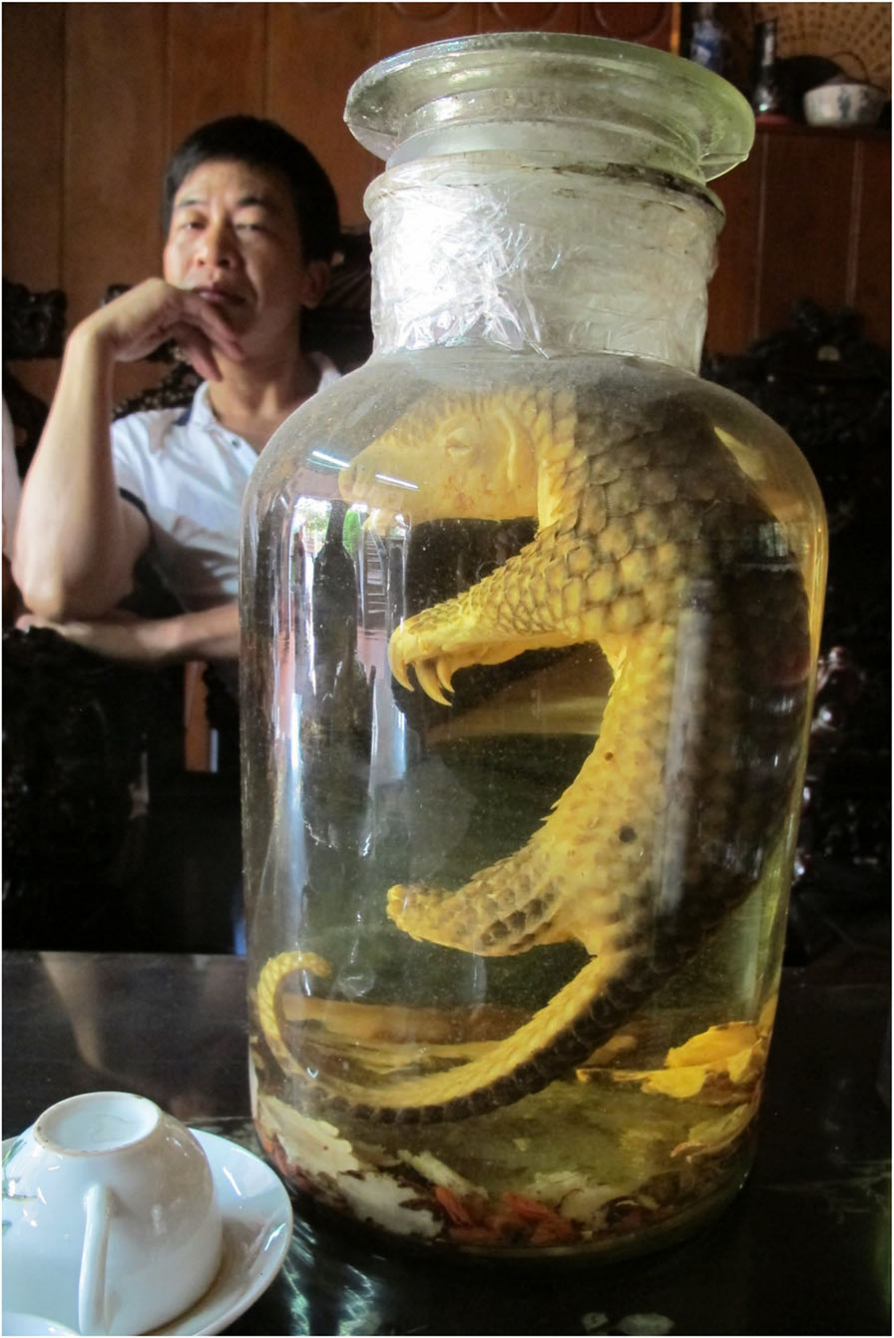
Pangolin wine (http://www.martinanthonyfletcher.com/pangolins-in-peril)

In China, pangolins are highly sought after for traditional medicine (Fig. 1) and as food [72]. This demand causes over-exploitation that, coupled with habitat loss, threatens the very survival of the species used. Pangolin scales are regarded as a medicinal panacea (like rhino horns, and like rhino horns they are made of keratin), and their meat is considered a delicacy. The demand for pangolins in China is met by an illegal trade that is lucrative and on the rise, lately attracting wildlife traffickers who used elephant ivory as their prime generator of profits. The demand for pangolin meat and scales, due to increasing conspicuous consumption by the Asian middle class, has driven pangolins to the verge of extinction [73]. From all forested corners of the tropics, pangolins are transported to Asian markets, where stressed and likely immune depressed pangolins are caged with many other species, and also with their own pathogens. This has emptied forests of pangolins: a steady decrease of pangolins, and wildlife in general, in African forests has been reported by local hunters and traditional healers in studies in Southwestern Nigeria [67] and in Cameroon [74].

The pangolin is prized as a delicacy in China, especially in the Southern and Eastern part of the country [75]. According to Challender et al. [76], this culinary practice is attested to by historical sources dating back to the 12th century CE: in present-day Jiangxi Province, Chinese pangolin meat was common street food during wintertime, cooked in lees from fermented rice wine. A popular recipe from the mountain village of Zhu Yu, dating back to the 16th century CE, consisted of curing pangolin meat in salt for two days before boiling it in water [77]. Nowadays, pangolin is served in high-end restaurants in urban cities, mostly in Anuhi, Fujian, Jiangxi, Guangxi, Yunnan, Guizhou, Guangzhou, Guangdong, and Guangxi provinces.

[75, 76, 78–80]. Once the order is placed, the animal may be hammered until it is unconscious and then slaughtered in front of the customers as a guarantee of the meat’s freshness. Some other time instead the animal is smuggled to the restaurant already dead and preserved in ice. Blood is drained and usually given to the customer to bring home. The dead animal is placed in hot water to remove the scales and the meat is cut into small pieces [81], which then may be boiled, stewed, braised, or steamed.

Chopped pangolin meat is usually stewed with Chinese wine, other meat including chicken or pork, and medicinal herbs such as *Ligusticum striatum*, *Tetrapanax papyrifer*, *Stemmacantha*, and *Akebia* spp. [82].

**Fig. 2:**
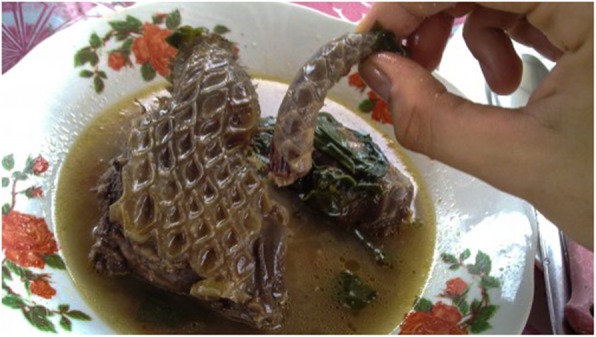
Pangolin soup (https://www.onegreenplanet.org/environment/where-have-all-the-pangolins-gone/)

In Shenzhen (Guangdong Province), pangolin meat is served in hot pot [81]. Pangolin meat is also an ingredient of “eight animal stew”, a dish made from animals like pangolin, swan, and snake simmered together for five hours, and a soup prepared with pangolin meat and caterpillar fungus (*Ophiocordyceps sinensis*) [83] (Fig. 2).

**Fig. 3:**
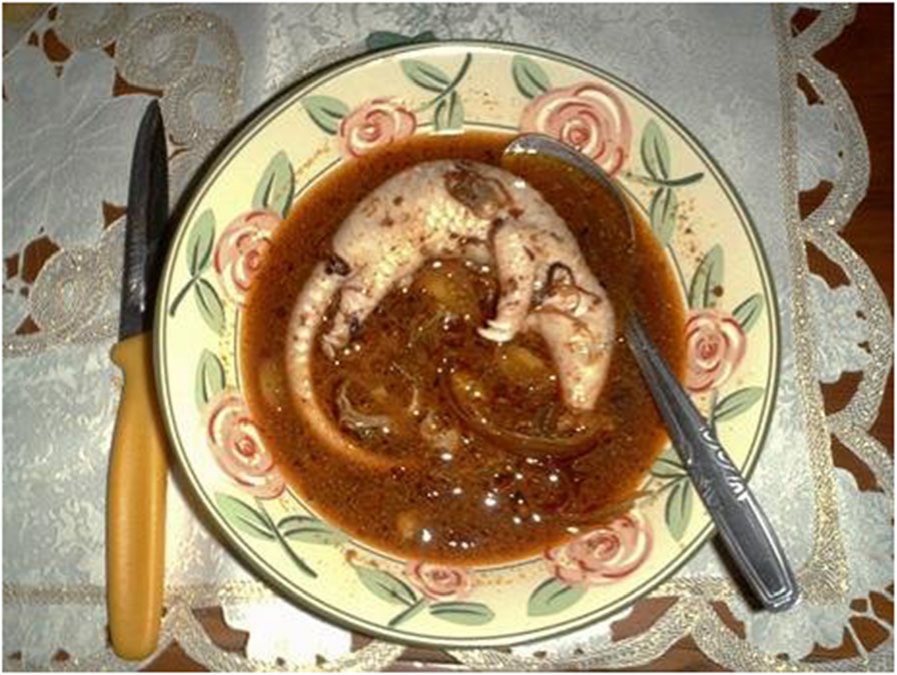
Pangolin fetus soup (https://allyouneedisbiology.wordpress.com/2015/04/20/pangolin-extinction/)

Several recipes including pangolin meat are prepared in Fujan gastronomy. In the western mountainous area, pangolin meat is steamed, simmered, and served/covered with a gelatinized sauce made with onion, soy sauce, ginger, Shaoxing wine, chicken soup, and Danggui (*Angelica sinensis* roots) [79]. A soup is also prepared by boiling the meat, which is served with pieces of pangolin tongue [84]. In the villages of the Yunnan–Guizhou Plateau (Yunnan and Guizhou provinces), a pangolin and chestnut stew is part of the local cuisine [80]. Besides meat, pangolin fetuses are eaten in soup (Fig. 3).

**Fig. 4:**
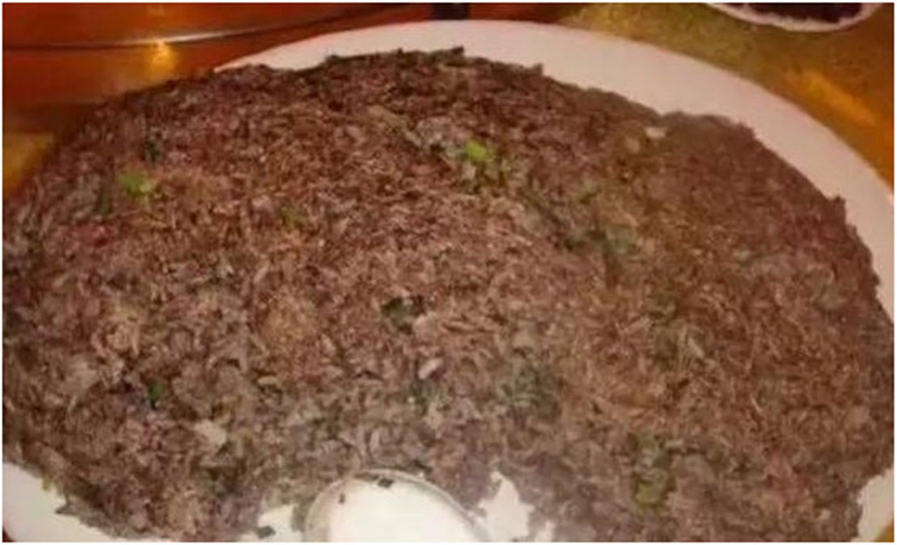
Pangolin blood rice (https://www.thatsmags.com/shenzhen/post/17691/shenzhen-woman-eats-pangolin-soup-enraging-netizens)

Moreover, baby pangolins are boiled in rice wine to brew a tonic and the blood is used as an ingredient in pangolin-blood fried rice (Fig. 4) [85].

## Self-regulating mechanisms mitigating potential overexploitation in TILEK systems

As subsistence-oriented populations are integrated into the global economy, processes of intensification and commodification of resources previously used for subsistence take place. These commodification processes often end up severing the links that existed between resource extraction and the carrying capacity and ecology of the surrounding environment. Populations lose their raw materials and spiritual attachment to their own restricted resource catchments, and these catchments become providers of both cash for hunters and highly-sought after products for global consumers. In the process, the same traditional knowledge, norms, and practices that have sustained a low-rate harvest of materially and culturally meaningful species change: while the knowledge and skills related to hunting and to the behavior of, for example, pangolins remain key to providing these animals to the market, the norms that regulated their harvest collapse under the pressure of demand, livelihood commodification, and the shift of decision-making from communities to individuals and outsiders. Indigenous and traditional knowledge, norms, and beliefs that regulate human access to different species in different places at different times are nonetheless central to biodiversity conservation [86]. Indeed, traditional knowledge, its nuanced understanding of ecological relationships, and the limits it sets to over-harvesting are of great importance for biodiversity conservation and for local livelihoods [87, 88], as well as being an attribute of communities with continuity in resource use practices.

By investigating the knowledge systems that different populations have in relation to the environment and its species, ethnobiology and ethnoecology help to understand and conceptualize the links between local populations, natural resources, and their management. Each use of a species does not have just a material significance, but rather it is embedded in cultural and social systems that give meaning to that use and put that meaning into the context of the wider ecology on which communities depend and about which have deep knowledge. When animals and their products are divorced from their cultural ecological context and commodified at the national and international level, then the place of these animals in the local culture and ecology becomes irrelevant if they do not contribute to cash generation and profit extraction. As seen repeatedly during the Anthropocene, the severing of the dynamic link (and its constraints) between human populations and the ecology of the places in which they live opens the way for all kinds of distortions, disruptions, and global threats, including the threat of pandemics.

One of the mechanisms through which populations and communities try to regulate access to and extraction of resources is through taboos. The enforcement of taboos may strike a dynamic balance between the biological and ecological characteristics of a species and its rate of extraction and use. This is often achieved through cultural and social mechanisms that may be effective as long as social and cultural integrity is not replaced with and substituted by commodified livelihoods. In this way, uses and traditions may lead to wildlife conservation, as shown in several studies [74, 89]. Culture and tradition regulate the use of certain species; the replacement of cultures and traditions with Western culture and a profit-based economic system breaks those regulations, with dire effects on the targeted species. In a study about taboos among rural communities of Cameroon, Bobo et al. [74] found that local culture regulates wildlife extraction and use through social norms and taboos. Four types of taboos that regulate resource extraction can be distinguished: (1) species specific, which regulate access (e.g., hunting, fishing, gathering) to specific wild species of ecological or cultural relevance (e.g., totem species); (2) habitat, which regulate (e.g., forbid during certain times of the year) access to specific habitats (e.g., sacred forests); (3) method, which regulate the culturally sanctioned time, place, means, and ways through which an activity (e.g., hunting) can be performed; and (4) segment taboos, which impose restrictions on the consumption of certain animals by certain social groups such as women or children [87, 90, 91]. Through taboos and social norms, resource-dependent communities regulate the rate of use of the species they depend upon for their survival, thus fostering resilience and cultural and social continuity.

Contemporary forms of wild animal extraction respond instead to the principles of intensification and maximization (versus optimization) of resource use for global trade and profit generation. This commodification of wild resources and their embedding in global commercial chains is sustained by a high demand for wild animals and their parts for conspicuous consumption by urban and high-income consumers, particularly in Asian countries.

## Disconnected consumers and the importance of awareness

With the disconnection taking place between consumers on the one hand, and producers, biodiversity and local ecologies on the other hand, the knowledge that consumers need is no longer, or not only, about the ways of processing, cooking, and eating foods, but also and importantly about the consequences that their decisions about what to eat have on distant livelihoods and ecologies. Several scholars have argued, in this respect, for the important role of consumer education in food habits and choices to reduce demand for prestige meat [92]. For example, shark fin soup is a preferred dish for ostentatious wedding ceremonies, birthday parties, and business meetings in China, and the demand for shark fins (often obtained through the practice of finning, which involves cutting the shark’s fin and throwing the shark back into the water) is pushing shark populations towards collapse [93, 94]. However, since about 2011, there has been an estimated 50–70% decrease in shark fin consumption in China, following many educational campaigns on the issue. In a survey about shark fin consumption conducted by WildAid, about 75% of the respondents did not even know the meat in the soup was from sharks, apparently because the name of the dish in Mandarin is “fish wing soup.” This is encouraging in relation to the importance of education. In the wake of the COVID-19 pandemic, the Chinese government has shut down wet markets all over the country and has begun a campaign of awareness concerning the importance of protecting wild species for collective health. The banning of wet markets, wildlife trade, and wildlife farming, without driving down the demand for wild meat, risks causing the trade to move underground, with a potentially even worse impact on commercialized species. Rather, demand can be reduced by informing and educating consumers about the consequences of their food desires and habits; there is no prestige in driving species to extinction. At the same time, there is the need to support alternative livelihoods for hunters, traders, and wildlife farmers if and when banning wet markets and wildlife trade. In the absence of alternative means of subsistence, any ban on wildlife trade and consumption will have a disproportionate effect on their livelihoods, pushing many of them into poverty and illegality [95]. Questions on how to alleviate poverty, and what outcomes this would have on bushmeat consumption, are nonetheless open to debate [96].

At the same time, as zoonotic diseases emerge not only from wildlife trafficking for human consumption, but also, as discussed, from the encroachment of human activities into forests as a result of land use changes and the expansion of intensive husbandry systems, and also from the disruption that these processes bring to the forest and the ecology of its species (including that of viruses and bacteria), and as these changes are an integral part of the Anthropocene, there is the need to rethink both our relationships with the rest of Earth’s community (materially and spiritually) and our global food system based on intensification and commodification, which creates profits for the few at the expense of everyone else and their health. Rethinking the global food system implies re-localizing food production, reconnecting it with the specific ecology of each place where food is produced, reconnecting producers and consumers, attuning each system to the local ecology of each place, creating value chains that empower all the stakeholders and not just a few at the expense of the many. Traditional and local knowledge, practices, norms, foods, and recipes would then again become tools of attuned engagement with the surrounding natural environment, rather than extrapolated elements of a commodified feeding frenzy.

A crucial role in this change can be played by food storytelling as well. The average conspicuous consumers buy the final product based on the story, not the animal itself (they can also be served a specially prepared chicken). One way to oppose that malpractice would be to widely acknowledge the illusion of the exclusiveness of such “wild foods” and to re-articulate the existing narratives.

In this respect, phenomena such as COVID-19 need to be framed within discourses that redefine the perceived boundaries between human and non-human, between what are considered cultural and natural realms. From this perspective, in the economy of wild foods, often presented as prestige dishes within global imaginaries of gastronomic exclusivity, the “wild” is loosing its significance, as the wild is not wild anymore. On the other hand, the same imaginary is undermining not only local economies, but also global health. Thus, the rhetoric of the wild is increasingly reducing spaces for wildlife as much as the livelihood of those who base their economy therein. In this sense, now, maybe more than ever, that *wilderness* yields the paradoxical result of making the already fuzzy boundary between domesticated and wild even more fragile.

## COVID-19 and the Anthropocene

We might eventually ask ourselves what the relationships between the Anthropocene and the COVID-19 pandemic are. Is COVID-19 a creature of the Anthropocene like climate change? The main traits of the Anthropocene, i.e., ecosystem and biodiversity loss, disrupted and turbulent ecologies, pervasive human activity, intensification of land use, commodification of traditional foods and knowledge, indeed also shape the conditions for the emergence of zoonotic diseases. The spread of zoonotic viruses in the last hundred years, more so in connection with attempts at wildlife farming, recalls what previously happened during the domestication process of livestock thousands of years ago. Continued contact between wild animal species and humans is known to be a source of zoonotic diseases. With increasing close contact during and after the domestication process, and with the increasing densities of human communities, zoonotic diseases like measles emerged at that time. The sanctioning of wildlife farming by the Chinese Government has probably improved the livelihoods and economic conditions of wildlife farmers, many of whom have been pushed out of the livestock sector and into wildlife farming at the forest’s hedge during the 1990s by the expansion of intensive livestock husbandry [97], but may have contributed in opening up an old Pandora’s Box. Many of the diseases that have plagued humans over the last several thousand years derived from our close relationships with our domesticated species. In the same way that the historical process of livestock domestication has brought us new diseases, is it possible that contemporary attempts at wildlife farming are leading us down the same path? Indeed, both processes stem from the intensification of human-animal relations (e.g., the reliance, close-proximity and handling by humans of selected species, or their trade) which leads down a path that facilitates the crossing of the species barrier by viruses present in reservoir and intermediate hosts.

## Lessons to learn: future ethnobiological research trajectories

Phenomena such as the COVID-19 pandemic are forcing ethnobiologists to readdress the schedule of their academic agendas and not only of their daily lives. This paper was drafted by authors who normally share the same physical space in a small university in NW Italy, but that at the moment can only work together and converse using online tools. COVID-19 is also requiring  us to readdress our teaching strategies, our ways of intellectually interacting within the scientific arena, and, even more importantly, our research paths.

This pandemic will force us to rethink not only our “classic” priorities in ethnobiology but also to envision new epistemological trajectories aimed at more effectively mitigating the mismanagement of natural resources that ultimately threats our and other beings' existence.

Moreover, field studies will be more difficult during and after this pandemic, and, nevertheless, more work will need to be done in the near future along the following lines:

- Historical studies on epidemics and other zoonotic diseases linked to ethnography-based ethnobiological and ethnomedical studies;
- New trends in the intensification of use and commodification of specific living creatures and their ecosystems for food, medicinal, or other purposes;
- Research on the self-regulating systems (including commons and communal goods) that local communities put in place to avoid overexploitation of specific resources in TILEK systems;
- Ethnozoological and ethnobotanical research linked to robust ethnoecological and/or cultural anthropological analyses of the contexts of use, possibly addressing diachronic and spatial dynamics (and not merely lists of used species);
- Human ecological studies on how access to natural resources happens and how it changes in response to changing socio-cultural-political contexts;
- Surveys on the rising of new elitist gastronomies and conspicuous consumption;
- Eco-semiotic works dealing with models for understanding how representations of natural objects are constructed and function;
- Political ecological research on how governance systems at different levels may impact or mitigate these intensification processes;
- Environmental philosophical work aimed at (re)defining the Anthropocene in times of insecurity.

The next few months will tell us more about how COVID-19 will have impacted the way in which we look at the relationships among living creatures, ecosystems, and human societies, and how our awareness of the value of the “webs of life” will influence our future studies and related reflections.

## Footnotes

1 In a hypothetical blame game, we could also blame cheap squid and farmed salmon consumers in high-income countries for the next West African epidemic stemming from bushmeat consumption.
